# Phenotypic dysregulation of microglial activation in young offspring rats with maternal sleep deprivation-induced cognitive impairment

**DOI:** 10.1038/srep09513

**Published:** 2015-04-01

**Authors:** Qiuying Zhao, Xiaofang Xie, Yonghua Fan, Jinqiang Zhang, Wei Jiang, Xiaohui Wu, Shuo Yan, Yubo Chen, Cheng Peng, Zili You

**Affiliations:** 1 School of Life Science and Technology, University of Electronic Science and Technology of China, Chengdu 610054, China; 2 State Key Laboratory Breeding Base of Systematic Research, Development and Utilization of Chinese Medicine Resources, Pharmacy College, Chengdu University of Traditional Chinese Medicine, Chengdu 6111376, China

## Abstract

Despite the potential adverse effects of maternal sleep deprivation (MSD) on physiological and behavioral aspects of offspring, the mechanisms remain poorly understood. The present study was intended to investigate the roles of microglia on neurodevelopment and cognition in young offspring rats with prenatal sleep deprivation. Pregnant Wistar rats received 72 h sleep deprivation in the last trimester of gestation, and their prepuberty male offspring were given the intraperitoneal injection with or without minocycline. The results showed the number of Iba1^+^ microglia increased, that of hippocampal neurogenesis decreased, and the hippocampus-dependent spatial learning and memory were impaired in MSD offspring. The classical microglial activation markers (M1 phenotype) IL-1β, IL-6, TNF-α, CD68 and iNOS were increased, while the alternative microglial activation markers (M2 phenotype) Arg1, Ym1, IL-4, IL-10 and CD206 were reduced in hippocampus of MSD offspring. After minocycline administration, the MSD offspring showed improvement in MWM behaviors and increase in BrdU^+^/DCX^+^ cells. Minocycline reduced Iba1^+^ cells, suppressed the production of pro-inflammatory molecules, and reversed the reduction of M2 microglial markers in the MSD prepuberty offspring. These results indicate that dysregulation in microglial pro- and anti-inflammatory activation is involved in MSD-induced inhibition of neurogenesis and impairment of spatial learning and memory.

The stress exposure in the prenatal period can eventually lead to multiple behavioral abnormalities in the adult offspring^1^. Sleep deprivation is a form of physical and emotional stressors during pregnancy, which is associated with several harmful consequences to the mothers and their children, and can damage the mother-infant relationship^2^. In rodent animals, maternal sleep deprivation (MSD) reduces adrenal weight and susceptibility to harmful agents^3^ and causes hypertension and renal abnormalities in pups^4^. Sleep restriction during pregnancy may cause behavioral and oxidative metabolic changes in male offspring^5^. The investigation revealed that more than two-thirds of pregnant women reported poor sleep quality and shortened sleep duration, especially in the third trimester^6^. However, little research has been done to directly examine how MSD alters the physiological and behavioral processes in offspring, and what underlies the alterations.

When a mother is deprived of sleep, widespread alterations in behavioral, cognitive, immune, and metabolic processes can be observed^7^, independent of hypothalamic-pituitary-adrenal (HPA) axis activation^8^. Maternal stress has a profound influence on fetal development and exerts long-time effects on health outcomes in offspring^9^,^10^. Recent research showed that pro-inflammatory cytokines, including interleukin 1 (IL-1) and IL-1 receptor, and markers of activated microglia, were elevated in the hippocampus and cerebral cortex of offspring after pregnant stress, suggesting an activation of the local inflammatory response^9^. Prenatal stress also alters microglial development and distribution in postnatal rat brain^11^. Fetal brain development is sensitive to changes in glial cell function^12^, in which permanently activated or “primed” microglia contribute to the long-lasting impairment of adult neurogenesis^13^.

Microglia constitute the first line of defense against pathological changes within the central nervous system (CNS) microenvironment^14^ and play important roles in various brain pathologies including psychiatric disorders^15^,^16^,^17^,^18^. Its activation and inflammatory cytokines secretion plays a central role as modulators of the neural stem cells niches in different processes, such as proliferation, differentiation, migration, and survival^19^. Microglial activation is often divided into two phenotypic profiles: the classical activation (M1) and the alternative activation/deactivation state (M2)^20^. Classically activated microglia may contribute to the reduction of neurogenesis and dysfunction of the neurotrophic system by releasing inflammatory mediators, including cytokines such as Tumor Necrosis Factor-α (TNF-α), interferon-γ (IFN-γ), IL-1β, and IL-6^21^,^22^. The alternative phenotype, sometimes called neuroprotective microglial phenotype, is important for antagonizing the inflammatory-induced damages in CNS. Once over-activated in embryonic period, microglia continue to be primed and influence neuron survival into adulthood^12^,^14^.

Minocycline is a derivative of tetracycline that exerts neuroprotective properties in neurodegenerative diseases^23^. It is a highly lipophilic molecule capable of crossing the blood-brain barrier^24^. The neuroprotective effect of minocycline has been shown to be mediated by inhibition of the proliferation and activation of microglia^22^,^23^. Thus, minocycline is a useful tool to investigate the mechanisms underlying microglial polarization and the pathogeneses of many diseases accompanied by microglial activation^22^,^25^. Previous studies from our laboratory indicated that MSD increased the number of Iba1^+^ (Ionized calcium-binding adaptor protein-1) cells and decreased hippocampal neurogenesis, induced imbalance between pro- and anti-inflammatory cytokines in the brain, and resulted in cognitive deficits and behavioral abnormalities in young offspring^26^. Nevertheless, the mechanism of microglial activation and dysregulation of cytokine signaling pathway involved in the precipitation of postnatal brain and behavioral pathology still remains to be explored. The purpose of this study was to investigate the effect of switching microglial phenotypes on cognitive function, neurodevelopment and inflammatory response in prepuberty offspring rats whose mothers were subjected to sleep deprivation.

## Results

### Minocycline administration improved MSD-induced changes in hippocampus - dependent spatial learning and memory of young offspring

**Figure 1:**
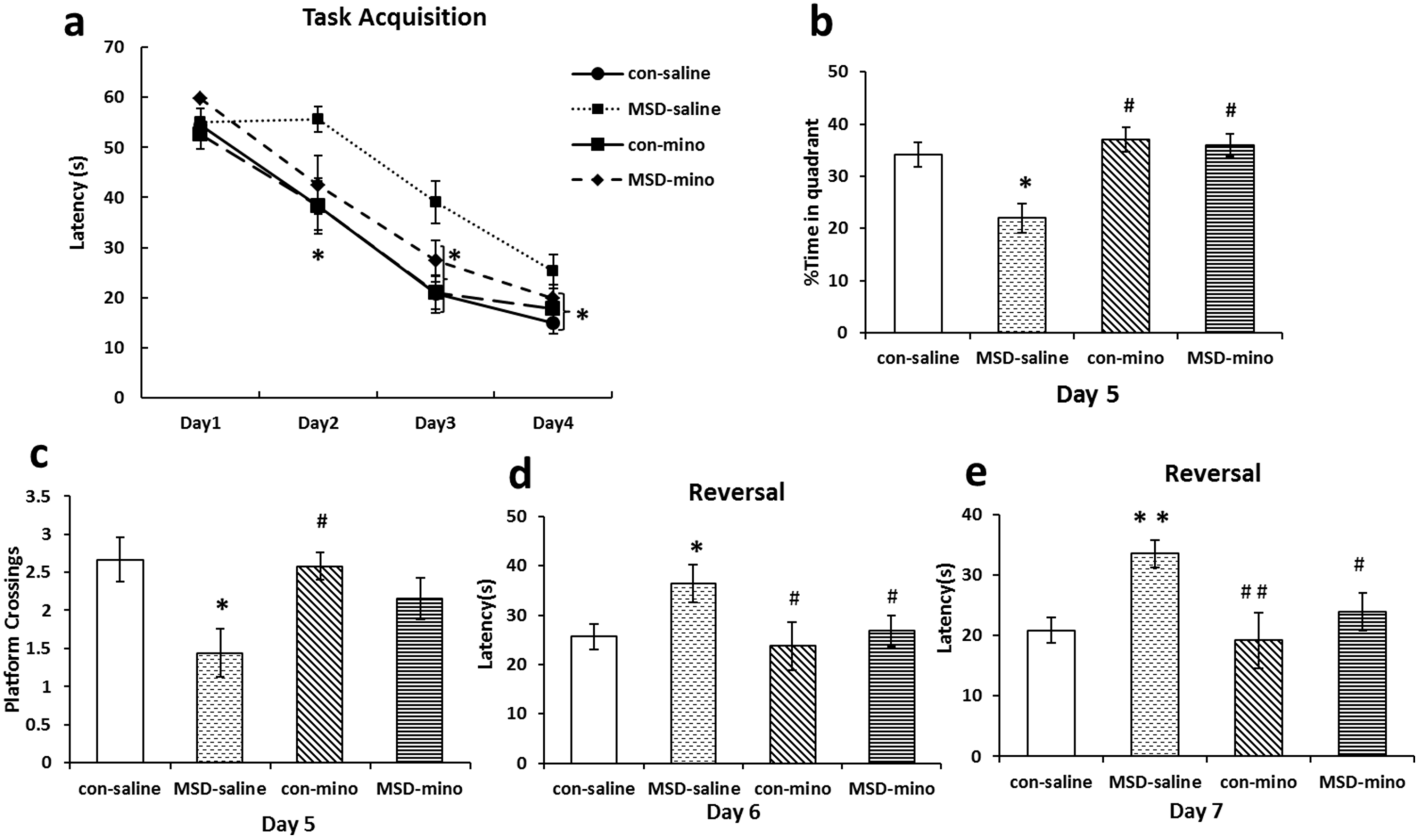
MSD impaired hippocampus-dependent spatial learning and memory of young offspring and impact of minocycline administration. During the task acquisition (a), the MSD-mino offspring spent a significantly fewer time than the MSD-saline rats to locate the hidden platform on day 3, and 4. On day 5, a probe trial was performed. MSD-mino offspring spent more time in the training quadrant (b) and passed over the original platform location more times (c). MSD-mino offspring spent less time than MSD-saline groups in reversal phases and restored to the con-saline prepuberty rats (d, e). * P < 0.05, ** P < 0.01 *vs*. the control-saline. *^#^ P* < 0.05, *^# #^ P* < 0.01 *vs*. with the MSD-saline. Values are the mean ± SEM.

Hippocampus-dependent spatial learning and memory was assessed using the MWM in young offspring on postnatal Day 21. The four groups did not differ significantly in latency to locate the platform on Day 1. The MSD-saline animals spent more time reaching the hidden platform than the control-saline and control-minocycline groups on Day 2–4. Nevertheless, after minocycline administration, the MSD group had a shorter latency than the MSD-saline group on Day 3–4 (Fig. 1a; day 1: *F*_(3, 26)_ = 1.844, *p* = 0.147; day 2: *F*_(3, 26)_ = 3.458, *p* = 0.021; day 3: *F*_(3, 26)_ = 4.600, *p* = 0.006; day 4: *F*_(3, 26)_ = 3.039, *p* = 0.038). When the platform was removed for the probe trial on Day 5, the MSD-saline group spent significantly less time in the training quadrant than other groups (Fig. 1b; *F*_(3, 26)_ = 3.54, *p* = 0.018). To further characterize memory deficits, we also recorded the number of platform crossings in a circular area circumscribing the original platform location, and found that the number of platform crossings was significantly smaller in the MSD-saline group than in control groups (Fig. 1c; *F*_(3, 26)_ = 6.45, *p* = 0.008). The MSD group after minocycline administration spent more time in the training quadrant than the MSD-saline group. The number of platform crossings in MSD-minocycline offspring was larger than in MSD-saline group. In the reverse trial, when the platform was placed in the quadrant opposite to the original, the latency to locate the platform increased in the MSD-saline group on Day 6 and Day 7 (Fig. 1d; *F*_(3, 26)_ = 3.44, *p* = 0.047), but decreased in the MSD group after minocycline administration (Fig. 1e; *F*_(3, 26)_ = 3.32, *p* = 0.048).

### Minocycline administration ameliorated MSD induced deficits in neurogenesis in hippocampus of young offspring

**Figure 2:**
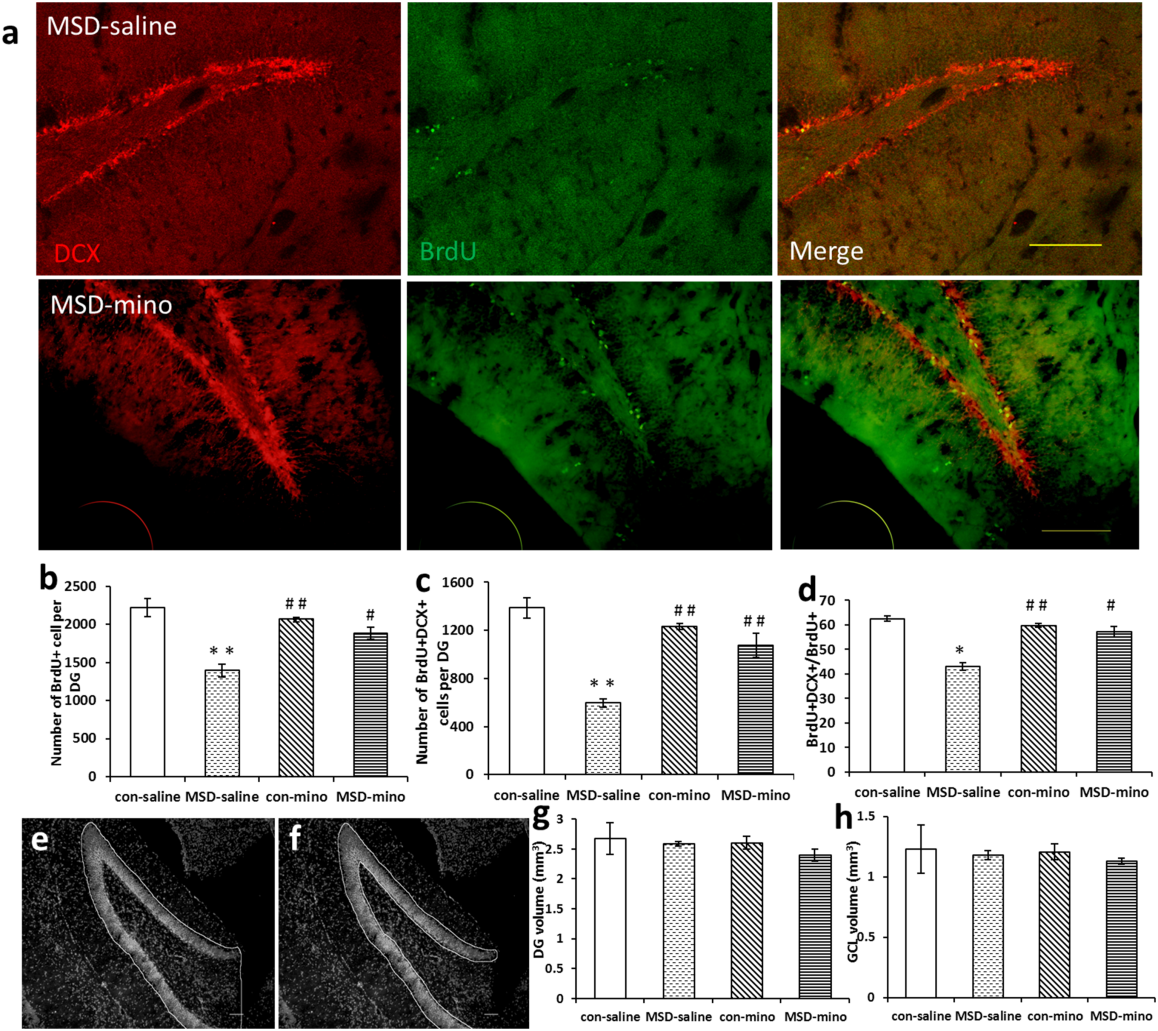
MSD induced changes in neurogenesis in hippocampus of young offspring and impact of minocycline administration. (a) Representative images of BrdU and DCX labeled cells in the DG of hippocampus, arrow represent the merged images. The total number of BrdU^+^ cells (b), the number of BrdU^+^/DCX^+^ cells (c) and the percentage of BrdU^+^/DCX^+^ out of all BrdU^+^ cells (d) were reduced in the MSD young offspring rats. After minocycline treatment, the total number of BrdU^+^ cells, the number of BrdU^+^/DCX^+^ cells and the percentage of BrdU^+^/DCX^+^ out of all BrdU^+^ cells were increased. Representative figures of the volumes of DG (e) and the GCL areas (f). (g) The volume of DG was not altered in young offspring rats. (h) MSD-mino did not change the volume of GCL subregion in young offspring. * P < 0.05, ** P < 0.01 *vs*. the control-saline. *^#^ P* < 0.05, *^# #^ P* < 0.01 *vs*. the MSD-saline. Values are the mean ± SEM. Scale bars: A: 20 μm; E, F: 10 μm.

BrdU^+^ and DCX^+^ cells indicated in the DG of hippocampus (Fig. 2a). The total number of BrdU-positive cells in the DG of hippocampus in the MSD-saline group was significantly smaller than that of the other three groups. After minocycline administration, however, the number of BrdU-positive cells increased in the MSD group (Fig. 2b; *F*_(3, 18)_ = 18.49, *p* = 0.001). The number of BrdU^+^/DCX^+^ cells decreased in the MSD group, but increased after minocycline injections (Fig. 2c; *F*_(3, 18)_ = 135.50, *p* < 0.001). While the ratio of BrdU^+^ DCX^+^/BrdU^+^ decreased in the MSD-saline group, it almost reached the control level after minocycline administration (Fig. 2d; *F*_(3, 18)_ = 46.18, *p* < 0.001).

DAPI stained sections were used to measure the volumes of neurogenesis regions in the offspring hippocampus. Fig. 2e outlines DG and Fig. 2f shows the subregion of GCL. No significant differences in the volumes of DG (Fig. 2g) and GCL areas (Fig. 2h) were identified between the MSD-saline and MSD-minocycline groups (Fig. 2g; DG: *F*_(3, 18)_ = 6.138, *p* = 0.625; Fig. 2h; GCL: *F*_(3, 18)_ = 4.565, *p* = 0.720).

### Minocycline treatment reduced microglial activation caused by MSD in hippocampus of young offspring rats

**Figure 3:**
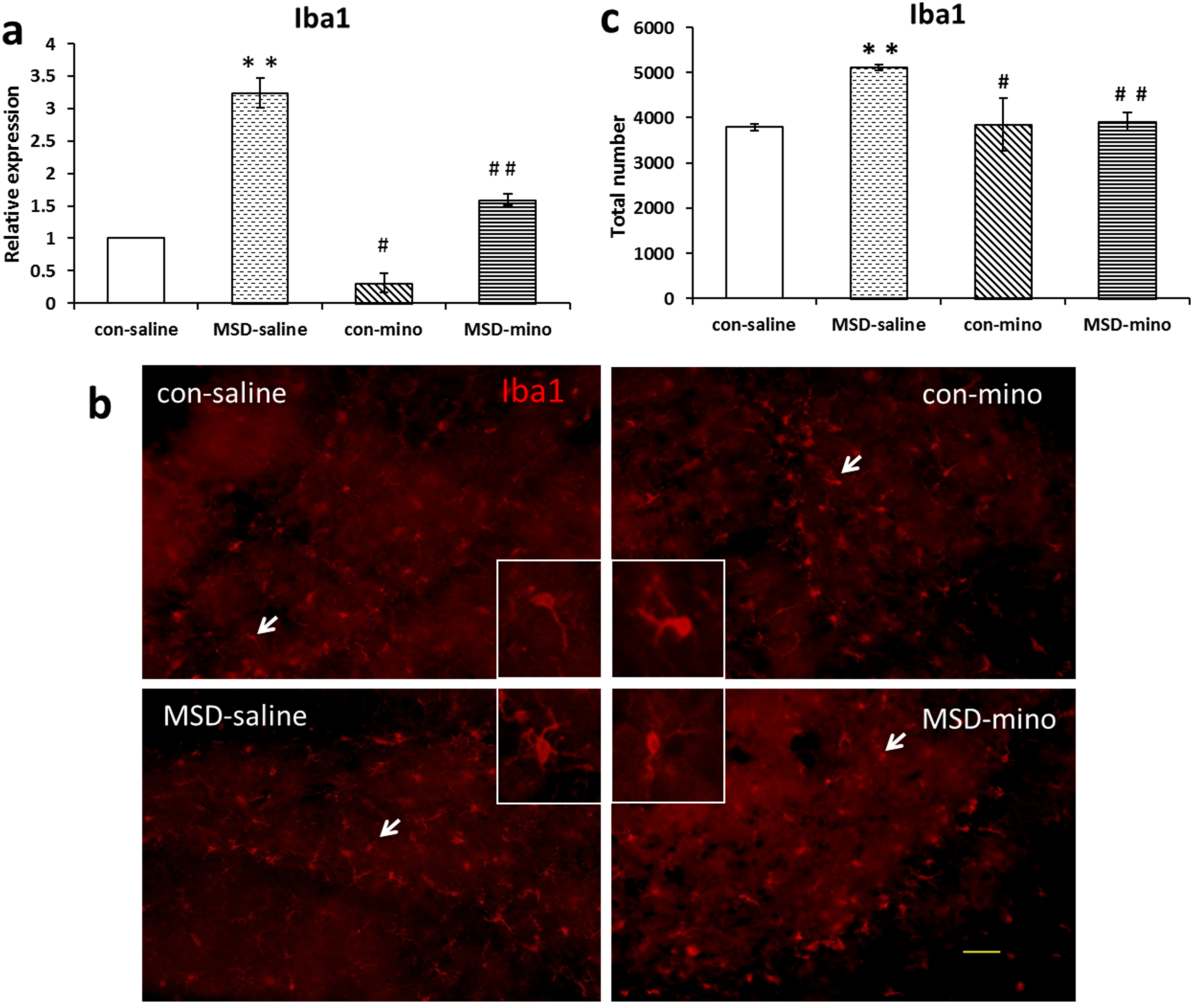
Minocycline treatment reduced microglia activation caused by MSD in hippocampus of young offspring rats. (a) Relative mRNA expression of Iba1 was increased after MSD, and the expression of Iba1 was decreased by minocycline treatment. (b) Representative example of microglia and its morphology in the DG of hippocampus. (c) The total number of Iba1^+^ cells also increased in the MSD-saline offspring rats, the microglia show large somas, short thick processes and a rounded amoeboid morphology, while the number decreased after minocycline injections and the microglial morphology returned to resting state. The arrows indicate typical microglia. * P < 0.05, ** P < 0.01 *vs*. the control-saline. *^#^ P* < 0.05, *^# #^ P* < 0.01 *vs*. the MSD-saline. Values are the mean ± SEM. Scale bars: 10 μm.

The expression of mRNA for Iba1 increased in the MSD-saline young offspring. After minocycline administration, the level of Iba1 was reduced (Fig. 3a; *F*_(3, 26)_ = 7.542, *p* = 0.002). The control-saline offspring exhibited no activation of microglia. The microglia in the MSD-saline offspring had large somas, short thick processes, and rounded amoeboid morphologies, known as hallmarks of activated microglial cells. The morphology of microglia in the MSD-minocycline was rod-shaped cell bodies with fine, ramified processes, a resting condition (Fig. 3b). Compared with MSD-saline group, the number of Iba1 positive microglia in the hippocampus of the MSD-minocycline group was significantly reduced (Fig. 3c; *F*_(3, 18)_ = 31.232, *p* = 0.005).

### Minocycline decreased the expression of M1 markers in the hippocampus of young offspring rats

**Figure 4:**
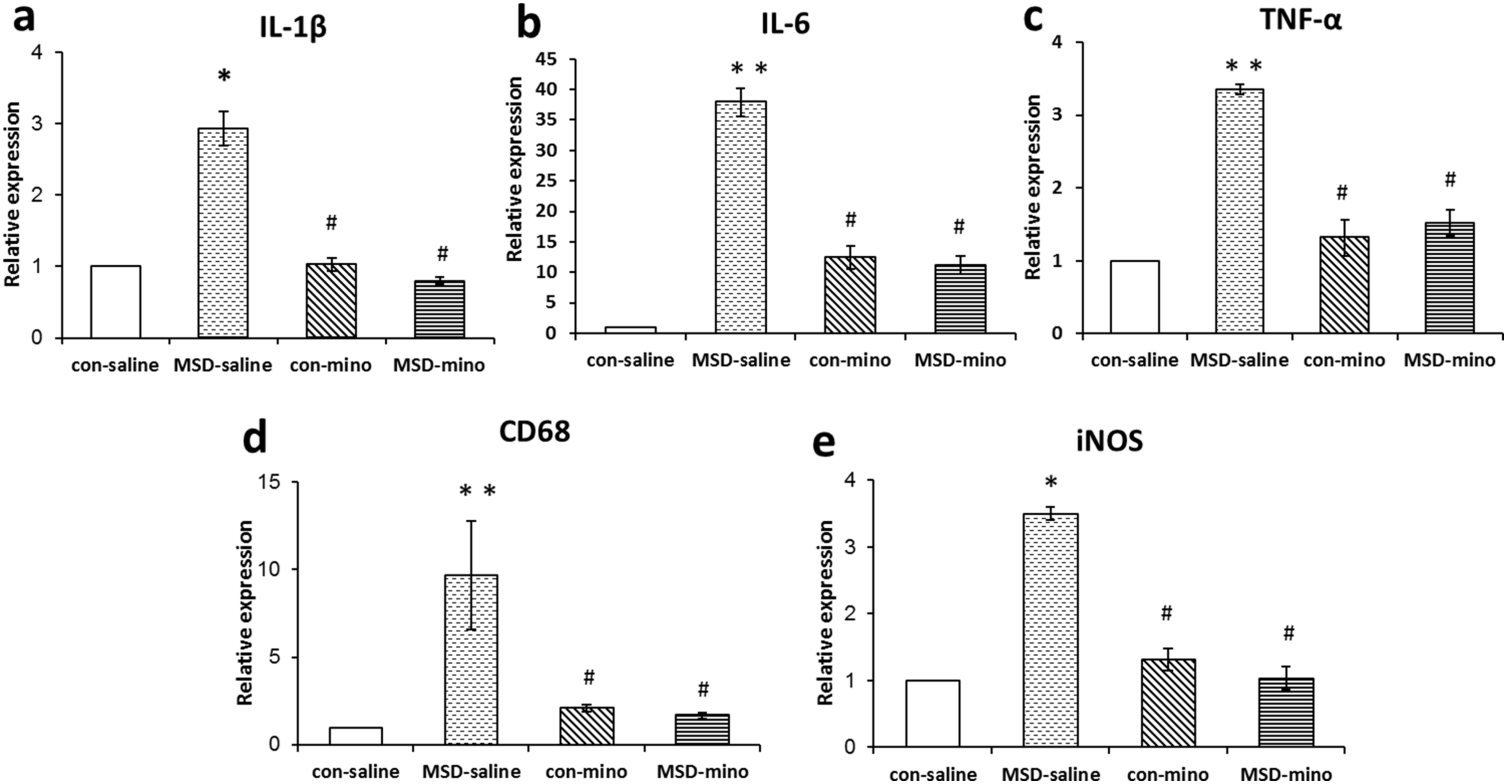
Minocycline decreased the expression of M1 markers after minocycline administration in the hippocampus of young offspring rats. The expression of M1 markers IL-1β (a), IL-6 (b), TNF-α (c), CD68 (d) and iNOS (e) increased in the MSD offspring. After minocycline injections, the levels of IL-1β, IL-6, TNF-α, CD68 and iNOS were lowered. * P < 0.05, ** P < 0.01 *vs*. the control-saline. *^#^ P* < 0.05, *^# #^ P* < 0.01 *vs*. the MSD-saline. Values are the mean ± SEM.

**Figure 5:**
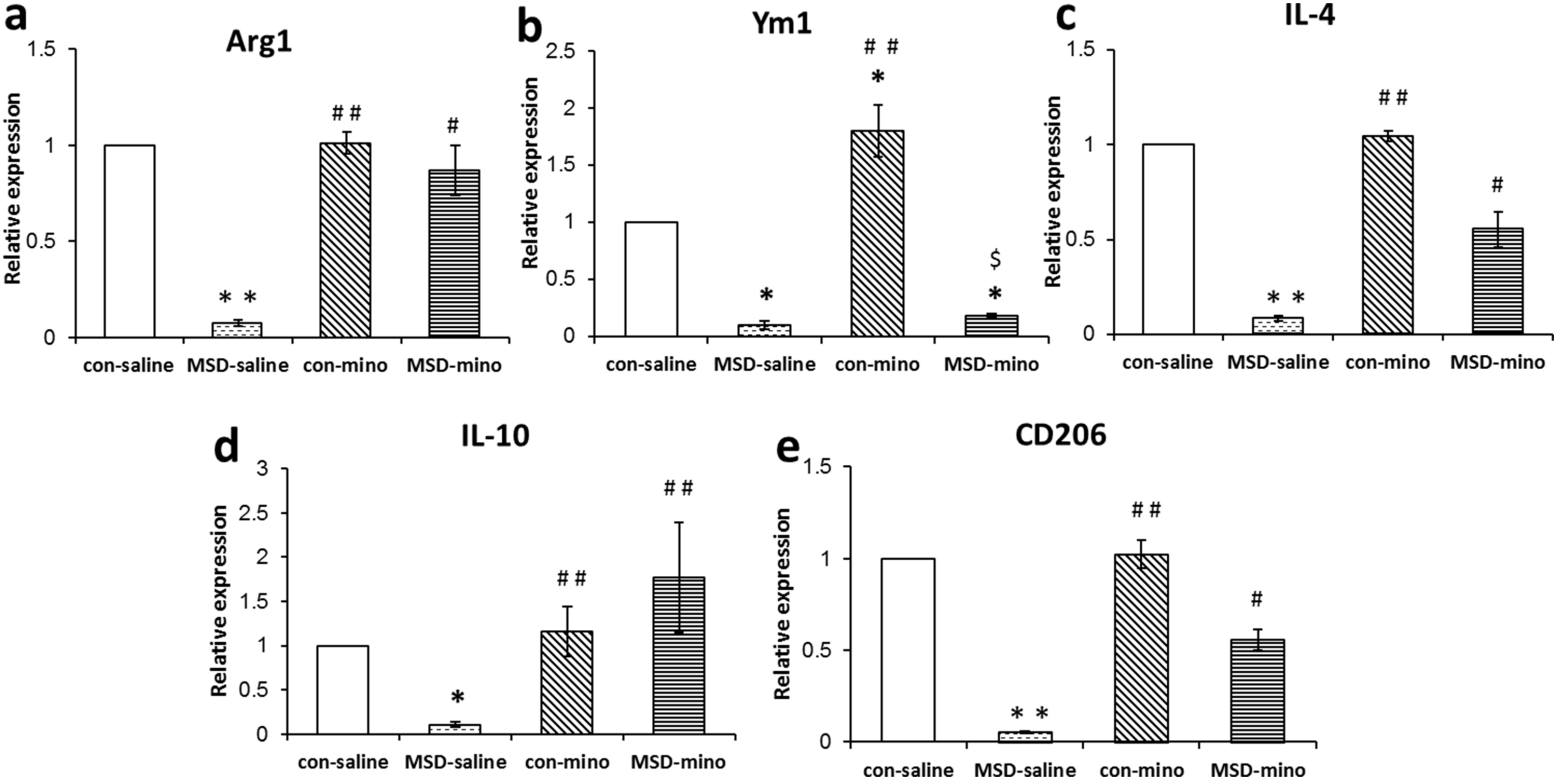
Minocycline enhanced the expression of M2 markers by minocycline treatment in the hippocampus of young offspring rats. The expression of M2 markers (Arg1, Ym1, IL-4, IL-10, and CD206) decreased in the MSD offspring rats. These decreases were improved by minocycline administration in the MSD offspring (Figs. 5a–5e). * P < 0.05, ** P < 0.01 *vs*. control-saline. *^#^ P* < 0.05, *^# #^ P* < 0.01 *vs*. MSD-saline. ^$^ *P* < 0.05 *vs*. con-mino. Values are the mean ± SEM.

The expression of M1 markers IL-1β, IL-6, TNF-α, CD68, and iNOS was increased in hippocampus of the MSD offspring. These increases were significantly attenuated by treatment with minocycline (Fig. 4a: *F*_(3, 26)_ = 4.573, *p* = 0.011; Fig. 4b: *F*_(3, 26)_ = 8.469, *p* = 0.001; Fig. 4c: *F*_(3, 26)_ = 9.012, *p* = 0.001; Fig. 4d: *F*_(3, 26)_ = 7.571, *p* = 0.015; Fig. 4e: *F*_(3, 26)_ = 6.202, *p* = 0.016). By contrast, the expression of M2 markers (Arg1, Ym1, IL-4, IL-10, and CD206) was reduced in the hippocampus of the MSD young offspring. After minocycline administration, the expression of Arg1, IL-4, IL-10, and CD206 was enhanced in the MSD offspring (Fig. 5a: *F*_(3, 26)_ = 6.965, *p* = 0.002; Fig. 5c: *F*_(3, 26)_ = 11.219, *p* = 0.001; Fig. 5d: *F*_(3, 26)_ = 11.867, *p* = 0.012; Fig. 5e: *F*_(3, 26)_ = 8.391, *p* < 0.001). However, Ym1 mRNA level was not changed by minocycline treatment in the MSD offspring (Fig. 5b; *F*_(3, 26)_ = 18.907, *p* < 0.001).

### Minocycline attenuated IL-6 and increased Arg1 protein expression derived from activated microglia in the hippocampus of young offspring rats

**Figure 6:**
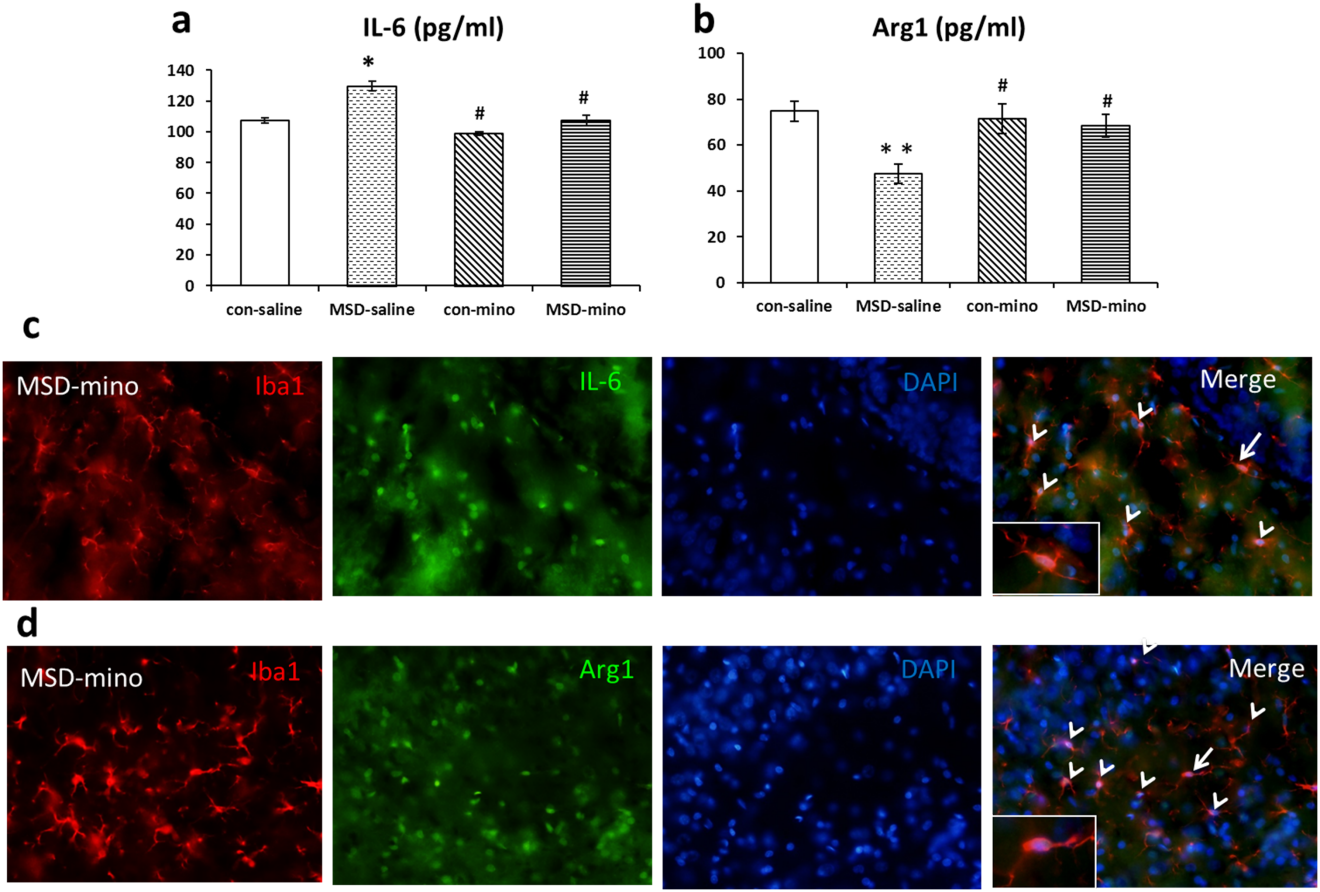
Minocycline altered IL-6 and Arg1 protein expression derived from activated microglia in the hippocampus of young offspring rats Hippocampal IL-6 protein level increased (a) and Arg1 (b) protein level decreased in the MSD-saline offspring. These changes were restored to the level of control-saline rats after minocycline treatment. Representative fluorescent images of the MSD-mino brain slices examined for Iba1 and IL-6 (c), Iba1 and Arg1 (d) immunoreactivity in the DG of hippocampus in the young offspring rats. The color of microglia is red, IL-6 and Arg1 are green. Arrowheads represent activated microglia, which released IL-6 (c) or Arg1 (d), arrows indicate fluorescent images of Iba1 and IL-6 or Arg1. * P < 0.05, ** P < 0.01 *vs*. control-saline. *^#^ P* < 0.05 *vs*. MSD-saline. Values are the mean ± SEM. Scale bars: 20 μm.

The expression of M1 marker IL-6 was higher and that of M2 marker Arg1 was lower after MSD in the hippocampus of prepuberty offspring. IL-6 mRNA level increased 37 fold in the MSD-saline offspring over the control-saline offspring, whereas Arg1 mRNA level was reduced by 90% (Figs. 4b and 5a). The IL-6 and Arg1 protein were detected using ELISA kits. In the hippocampus, the expression of IL-6 was enhanced after MSD compared with the control. The increase was attenuated by minocycline treatment (Fig. 6a: *F*_(3, 26)_ = 9.780, *p* = 0.006). The quantity of Arg1 protein increased in the MSD-minocycline group compared to the MSD-saline group (Fig. 6b: *F*_(3, 26)_ = 8.751, *p* = 0.007). IL-6 and Arg1 were generated from microglia of the MSD-minocycline group in the hippocampus of offspring rats (Fig. 6c, 6d).

## Discussion

Our data provide evidence that microglia play a pivotal role in modulating the impact of MSD on hippocampus neuronal activity and hippocampus-regulated behavior in the offspring. Minocycline can dampen MSD-induced microglial activation and hippocampal neurogenesis impairment, and also reverse the spatial learning and memory deficits in the MSD young offspring. The neuroprotective effect of minocycline in MSD offspring rats was related to switching the pro-inflammatory microglial response to anti-inflammatory microglial phenotype.

Stress during pregnancy has lasting effects on neurodevelopment and increases vulnerability to psychopathology in offspring^13^,^27^. In this study, we investigated the effects of sleep deprivation during pregnancy on the neurogenesis of the prepuberty male offspring. The total numbers of BrdU^+^ cells and BrdU^+^/DCX^+^ cells in MSD offspring were smaller than that of the control. This showed that hippocampal neurogenesis is reduced in the DG in young rats by MSD, which conforms with the findings of our previous research^26^. These results support the concept that prenatal stress can induce lasting and profound changes in the offspring, potently inhibiting neurogenesis^10^. The low level of neurogenesis was likely due to the established stress hyper-responsiveness, including the inflammatory response or neurodegeneraion^1^,^28^. We observed the suppressed neurogenesis in MSD offspring restored by intraperitoneal (i.p.) administration of minocycline, which was associated with specific inhibition of microglial activation^29^. However, no significant differences were identified in the volumes of hippocampus neurogenesis regions in the MSD-minocycline offspring. In addition to new neurons, other factors such as decreases in the survival of neurons, alterations in the somatodendritic, axonal, and synaptic components and glial changes are also corrected with hippocampal volume loss^30^.

Our behavioral studies with the young offspring rats that were prenatally stressed using the MWM tasks demonstrated impairment of spatial working and memory, which requires a fully functional hippocampus^31^. As shown in Fig. 1, the MSD-minocycline offspring spent significantly less time than the MSD-saline groups locating the hidden platform during the task acquisition and the reverse trial, spent more time in the target quadrant and they had a larger number of platform crossings than the MSD-saline offspring in the spatial exploration test. This indicates a deficit reversed in hippocampus-dependent spatial learning and memory in the MSD-minocycline offspring rats. In rodents, the hippocampus has long been recognized as a critical structure for encoding spatial information^32^. The experience-dependent modulation of neurogenesis in DG is involved in learning and memory^33^. MSD-induced impairment of hippocampus-dependent behavioral tasks was connected to inhibition of hippocampal neurogenesis in the offspring rats. According to hippocampal neurogenesis, the spatial learning and memory of MSD offspring were improved after anti-inflammatory treatment with minocycline. It appeared that the anti-inflammatory property of minocycline was responsible for the behavioral effects^25^.

Although Iba1 is constitutively expressed by microglia, it is only moderately expressed in quiescent microglia, but strongly expressed in response to activating stimuli^17^. MSD significantly increased the expression of Iba1 on mRNA level and the density of Iba1 positive cells in the offspring rats, as indicated by immunohistochemistry. We have shown in our previous research that the morphological features of microglia were large somas, short thick processes, and a rounded amoeboid in the offspring of MSD^26^. This suggests that sleep deprivation in pregnant dams could induce microglial activation in the prepuberty offspring, similar to prenatal immune challenges^9^. The pro-inflammatory status is considered a risk factor for development of behavioral alterations and cognitive deficits in animals and humans^11^,^34^. Minocycline can reduce microglia activation, which was demonstrated in this experiment by the down-regulation of mRNA expression of Iba1 and the reduction in the number of immunolabelling Iba1^+^ cells. The results indicated that the improvements of MWM behaviors and neurogenesis by minocycline treatment in the young offspring rats were related to the inhibition of activated microglia.

Insufficient sleep and poor sleep quality have pro-inflammatory effects^35^, which are independent of nonspecific consequences of sleep deprivation procedures^4^,^36^. In this study, the multiple-platform method was used to avoid the stresses of activity restriction^37^. The pro-inflammatory effect of sleep disturbance during pregnancy is a risk factor for embryonic developments through maternal-placental-fetal inflammatory pathways^38^. Intrauterine inflammatory exposure may induce microglial changes and mediate programming of neuroinflammatory processes in offspring^1^,^9^. Although cytokines can be produced by astrocytes, endothelial cells, even neuron^39^, microglia are the primary source for inflammatory mediators. The expression of pro-inflammatory factors such as IL-1β, IL-6, TNF-α, and iNOS was increased in the hippocampus of young rats whose mothers experienced sleep deprivation. Meanwhile, the expression of anti-inflammatory cytokines IL-4 and IL-10 was reduced in the MSD prepuberty rats. Opposing the deleterious effects of pro-inflammatory cytokines, anti-inflammatory cytokines such as TGF-β^40^ and IL-10 41 have pro-neurogenic effects on adult neural stem/progenitor cells. The dysregulation between pro- and anti-inflammatory cytokines plays an important role in the impairments of neurogenesis^42^.

Studies on peripheral macrophages have led to the identification of distinct activation profiles, referred to as classical (M1) and alternative (M2) activation states^43^. These are characterized by different markers being expressed and different functions of the cells. On one hand, the expression of M1 markers (IL-1β, IL-6, TNF-α, CD68 and iNOS) gradually increased in the MSD group but not in the control groups. On the other hand, the expression of M2 markers (Arg1, Ym1, IL-4, IL-10, and CD206) was reduced in the MSD-saline group. Our data suggested that the expression of pro-inflammatory cytokines was induced and resulted in M1 microglial activation in the young offspring rats after MSD. Stress induced HPA axis hyperactivity can affect microglial activation and phenotypes, followed by neuroinflammatory outcomes^44^. These results support the concept that prenatal stress can induce long-term pro-inflammatory effects and classical microglial activation or M1 phenotype^9^. With regard to microglial phenotypes, we paid special attention to the current study to the alternative activation of microglia in the young offspring whose mothers were exposed to sleep deprivation. In contrast to the enhanced M1 microglial markers, the expression of M2 markers including Arg1, Ym1, IL-4, IL-10, and CD206 (Fig. 5) were reduced in the MSD offspring rats. Numerous studies have concluded that M2 microglia was neuroprotective while M1 microglia were neurotoxic^21^. M1 microglia may contribute to the deficit of hippocampal neurogenesis and related behaviors by releasing inflammatory mediators, including cytokines such as TNF-α, IL-1β and IL-6^20^,^22^, which will ultimately cause local inflammation and neurodegeneration^45^. With minocycline treatment, the M2 markers in MSD offspring were restored to the level of the control rats. The effects of minocycline on microglial activated phenotypes were consistent with its pro-neurogenic effect and neurobehavioral actions in young offspring rats. Results indicated that MSD caused deficits of hippocampus-dependent behavior and reduction of hippocampal neurogenesis in the offspring rats, which strongly correlated with microglial activation phenotype in young offspring rats.

Microglia, the brain's resident innate immune cells, are thought to derive from myeloid precursor cells that infiltrate the central nervous system via local blood vessels during embryonic and early postnatal life^14^. In the rat, microglia are first seen between embryonic Days 12–14, and then a rapid increase in the number of microglia during late trimester of fetal development period was observed^11^. Microglia are critical for early brain development and can respond vigorously to stress^12^. The third trimester appears to be a particularly sensitive period to inflammatory challenge^16^. Once prenatally challenged, the long-lived microglia are maintained in an activated or primed state into adulthood^46^,^47^. Our previous results demonstrated that microglial activation induced by late-stage pregnancy stress could last until postnatal day 21^26^. Microglia constitute the major niche^19^ and play a key role in controlling multiple steps of neurogenesis^29^. Prenatal stress induces microglia into a preponderant M1 cytotoxic phenotype in offspring rats^11^,^48^. Dysregulated microglial activation leads to the imbalance of pro- and anti-inflammatory cytokines^42^,^48^. The pro-inflammatory cytokines in CNS can damage neural cells directly or indirectly. The pro-inflammatory cytokines IL-1β, IL-6, TNF-α, and iNOS were increased and anti-inflammatory cytokine IL-4 and IL-10 was reduced after MSD (Fig. 4). It has been shown that the activation of microglia is associated with a reduction of new neurons in the rodent hippocampus, mainly due to the decreased survival of the new neurons^28^. Minocycline suppressed the production of pro-inflammatory molecules in the MSD prepuberty offspring, improved hippocampus-dependent spatial learning and memory, and promoted hippocampal neurogenesis. Minocycline exerts its neuroprotective effect by inhibiting the microglial polarization into M1, facilitating M2 activation^22^.

Minocycline is a semisynthetic tetracycline that exerts anti-inflammatory effects completely separate from its antimicrobial actions^49^. Studies suggest that it provides neuroprotection because it can cross the blood-brain barrier and inhibit the proliferation and activation of microglia^23^,^24^. Minocycline has neuroprotective qualities in animal models of neurodegenerative diseases and has been evaluated in clinical trials for a number of neurodegenerative or autoimmune diseases which affect the CNS, including stroke, Parkinson's disease, Huntington's disease, and multiple sclerosis^24^,^25^,^50^. These neuroprotective activities of minocycline have been attributed to its inhibitory effects on microglial activation^47^,^51^. This study indicated that microglial M1 suppressors such as minocycline could have potential benefit for behaviors and neurogenesis of offspring whose mothers experienced sleep deprivation during pregnancy.

In this study, we report for the first time that anti-inflammatory treatment can counteract neuroinflammatory responses and ameliorate cognitive impairment caused by MSD in offspring. Our study indicates that microglia play a significant role in determining neuronal proliferation and survival, which are responsible for the impairment of spatial learning and memory of young offspring rats in MSD model. The M1 microglial activation profiles with prominent pro-inflammatory cytokines are relevant to neural defense and neuropathology. Minocycline inhibit M1-biased microglial response and have beneficial effects in the young offspring rats whose mothers experience sleep deprivation during pregnancy.

## Methods

All experimental procedures were approved by the Institutional Animal Care and Use Committee at the University of Electronic Science and Technology of China. The methods were carried out in accordance with the approved guidelines.

### Animals

Adult male and female Wistar rats (three months old) were purchased from Chengdu Dossy Biological Technology Co., Ltd. (Chengdu, China), kept individually under standard housing conditions (12/12 h light/dark phase, lights on at 7:00 A.M., humidity 55 ± 5%, temperature 23–25°C), with *ad libitum* access to food and water, and habituated to the animal facilities for one week. The female rats were subjected to a timed mating procedure as described previously^26^. The first day of pregnancy was defined as gestational day (GD) 1.

### Sleep Deprivation

Sleep deprivation was performed using modified multiple-platforms method on late trimester (GD 18). Pregnant Wistar rats (n = 25) were placed individually in the experimental device in previously described^26^. All platform exposure began at 10:00 a.m. and ended after 72 h when the pregnant rats were put back into their home cages. All rats had free access to water and food throughout the experiment.

### Minocycline treatment

After weaning (21 days old), 2–3 male offspring were selected from each mother group. The male prepuberty offspring from non-MSD and MSD were given i.p. injections of saline and minocycline (50 mg/kg; Sigma-Aldrich, St. Louis, MO, USA) twice daily (8:00 a.m. and 8:00 p.m.) for the first two days, and then i.p. injections once daily at 8:00 a.m. for the following three days. Minocycline dose was selected according to the established dose regimen^52^. Minocycline was diluted in saline. On the second day after five days of injections, Morris Water Maze, Immunohistochemistry, ELISA and Real-Time PCR were performed among offspring. To avoid the effects of hormonal variation in females, only male offspring were used for the study. The offspring were divided into four groups: control-saline (n = 27), MSD-saline (n = 27), control-minocycline (n = 26), and MSD-minocycline (n = 27).

### Morris Water Maze

The prepuberty offspring rats (21 days old) were to find a hidden platform (9 cm in diameter) 2 cm under the water surface in a pool (1 m in diameter), four trials *per* day for four consecutive days as previously described^26^. All MWM testing was performed between 9 a.m. and 2 p.m. The MWM was performed by a researcher blinded to the identity of groups. The young offspring rats of the control-saline (n = 8), MSD-saline (n = 8), control-minocycline (n = 7), and MSD-minocycline (n = 8) groups were used in the MWM experiment.

### Immunohistochemistry

Offspring rats (21 days old) from the control-saline (n = 5), MSD-saline (n = 5), control-minocycline (n = 5), and MSD-minocycline (n = 5) groups were measured in the immunohistochemical analysis for neuronal proliferation and microglial morphology. Prepuberty offspring rats were given one injection of Bromodeoxyuridine (BrdU) (50 mg/kg, i.p., Sigma, USA) at 8:00 a.m. Two hours after injection, the rats were anesthetized with pentobarbital sodium and perfused transcardially with saline, followed by 4% paraformaldehyde in phosphate-buffered saline (PBS). Brains were carefully removed and postfixed with 4.0% paraformaldehyde for 48 h and in 30% sucrose for 24 h. Coronal sections (35 μm thick) were cut using a sliding vibratome (CM1900; Leica Microsystems, Wetzlar, Germany) and collected in PBS with 0.03% sodium azide. Six sequential slices were placed into each well of a 12-well plate and stored at 4°C. For BrdU staining, the sections were permeabilized with Triton X-100 (0.5% in PBS) for 20 min, and pretreated by incubation in 2 N HCl for 30 min at 37°C, followed by two washes with 0.1 M borate buffer (pH 8.5) for 10 min each. The slices were blocked in 10% donkey serum for 2 h, incubated with primary antibodies overnight at 4°C, and then incubated with secondary antibodies. For Doublecortin (DCX), Iba1, IL-6 and Arginase1 (Arg1) labeling, but not HCl and borate buffer incubation, the other procedures were the same as BrdU staining. The primary antibodies were mouse anti-BrdU (1:500; Cell Signaling Technology, USA), goat anti-DCX (1:400; Santa Cruz, USA), goat anti-Iba1 (1:400; Abcam, UK), mouse anti-IL6 (1:500; Abcam, UK), and rabbit anti-Arg1 (1:200; Abcam, UK). The secondary antibodies were Alexa Fluor 488-conjugate donkey anti-mouse (1:300; Jackson ImmunoResearch, USA), Alexa Fluor 488-conjugate donkey anti-rabbit (1:300; Jackson ImmunoResearch, USA), and Alexa Fluor 594-conjugate donkey anti-goat (1:300; Jackson ImmunoResearch, USA).

To quantify the proliferating cells and microglia, every sixth section from one animal containing hippocampus was selected and immunolabelled with antibodies. The resulting numbers were multiplied by 6 to estimate the total number *per* Dentate Gyrus^53^. Iba1 was used to evaluate the microglial activated phenotype according to Iba1 positive stain and cell morphology. Positive cells were manually counted under a 40× objective fluorescence microscope (Olympus BX51). Photomicrographs were saved as TIF files and quantitatively analyzed using the cell counter of Image J software (version 1.45J; National Institutes of Health, Bethesda, MD, USA).

To estimate the volumes of Dentate Gyrus (DG) and Granule cell layer (GCL), which are two different neurogenesis regions of hippocampus, we stained every sixth section per animal using 4′-6-diamidino-2-phenylindole (DAPI), following the Cavalieri principle. The volumes were measured using software ImageJ, version 1.45j (Fig. 2e and 2f). The volumes were computed by multiplying each section area by thickness of brain slice and number of sections^54^.

### ELISA

Hippocampal tissues were dissected, flash-frozen in liquid nitrogen, and stored at −80°C before use. The frozen lysates were homogenized and centrifuged at 1,000 × g for 30 min. Supernatants were applied to detect the expression of IL-6 and Arg1. The levels of IL-6 and Arg1 were quantified using ELISA kits (Rapid Bio, USA and BD, USA) according to the manufacturer's protocol. The detection limit for IL-6 and Arg1 were 1 pg/ml. The amount of IL-6 and Arg1 were expressed as the ratio of IL-6 and Arg1 to the total soluble protein content (pg/mg). Young offspring rats of the control-saline (n = 6), MSD-saline (n = 6), control-minocycline (n = 6), and MSD-minocycline (n = 6) groups were used in the ELISA test.

### Real-Time PCR

Male prepuberty offspring rats [control-saline (n = 8), MSD-saline (n = 8), control-minocycline (n = 8), and MSD-minocycline (n = 8)] were sacrificed by decapitation, and their hippocampus was quickly removed using aseptic technique, placed in sterile tubes placed on dry ice. Total RNA was extracted using Trizol reagent (Invitrogen Life Technologies, USA) according to the experiment's protocol. The RNA samples were suspended in 30 μl nuclease-free water. Then 5 μg of total RNA were reverse transcribed using the First Strand cDNA Synthesis Kit (Invitrogen Life Technologies, USA). The cDNA was stored at −20°C. All PCR reactions were operated under the following conditions: initial 98°C for 2 min followed by 38 cycles at 98°C for 2 s and 60°C for 10 s (Bio-Rad CFX 96). Each sample was tested in triplicate. Threshold amplification cycle number (C_T_) was determined for each reaction within the linear phase of the amplification plot, and relative gene expression was determined using the 2 ^−ΔΔCt^ method^55^. The values were normalized against the housekeeping genes GAPDH. Primer sequences were as follows: TNF-α, 5′-CATCTTCTCAAAATTCGAGTGACAA-3′, 5′-GGGAGTAGACAAGGTACAACCC-3′; IL-1β, 5′-CCCTGCAGCTGGAGAGTGTGG-3′, 5′-TGTGCTCTGCTTGAGAGGTGCT-3′; IL-6, 5′-TCTTGGGAC-TGATGCTGGTG-3′, 5′-CAGAATTGCCATTGCACAACTC-3′; CD68, 5′-CCACAGGCAGCACAGTGGACA-3′, 5′-TCCACAGCAGAAGCTTTGGCCC-3′; iNOS, 5′-GCCAAGTTTGAGGTCAACAACCCA-3′, 5′-CCCACCCCGAATCAGCAGCG-3′; CD206, 5′-AGTTGGGTTCTCCTGTAGCCCAA-3′, 5′-ACTACTACCTGAGCCCACACCTGCT-3′; IL-4, 5′-GGTCTCAGCCCCCACCTTGC-3′, 5′-CCGTGGTGTTCCTTGTTGCCGT-3′; IL-10, 5′-AATTCCCTGGGTGAGAAGCTG-3′, 5′-TCATGGCCTTGTAGACACCTTG-3′; Arg1, 5′-TCACCTGAGCTTTGATGTCG-3′, 5′-TTCCCAAGAGTTGGGTTCAC-3′; Ym1, 5′-ACCCCTGCCTGTGTACTCACCT-3′, 5′-CACTGAACGGGGCAGGTCCAAA-3′; Iba1, 5′-CTGGGAGATGTGAATGGAG-3′, 5′-ACTGATGCTGGCTACTGATG-3′; GAPDH, 5′-ATGACCCCTTCATTGACCTCA-3′, 5′-GAGATGATGACCCTTTTGGCT-3′.

### Statistical Analysis

All data were presented as mean ± SEM. The results were analyzed with repeated measures analysis of variance. Differences were considered significant when p < 0.05. Analyses were conducted using SPSS for Windows® v.17 (SPSS Inc., Chicago, USA).
